# The Influence of Alkali-Resistant MiniBars™ on the Mechanical Properties of Geopolymer Composites

**DOI:** 10.3390/ma18040778

**Published:** 2025-02-10

**Authors:** Gabriel Furtos, Codruta Sarosi, Marioara Moldovan, Kinga Korniejenko, Michał Łach, Viorel Ungureanu, Leonard Miller, Iveta Nováková

**Affiliations:** 1 Raluca Ripan Institute of Research in Chemistry, Babes-Bolyai University, 400294 Cluj-Napoca, Romania; codruta.sarosi@gmail.com (C.S.); mmarioara2004@yahoo.com (M.M.); 2 Faculty of Material Engineering and Physics, Cracow University of Technology, 31-864 Cracow, Poland; kinga.korniejenko@pk.edu.pl (K.K.); michal.lach@pk.edu.pl (M.Ł.); 3 Department of Steel Structures and Structural Mechanics, Civil Engineering Faculty, Politehnica University Timisoara, 300224 Timisoara, Romania; viorel.ungureanu@upt.ro; 4 Technical Science Academy of Romania, 010413 Bucharest, Romania; 5 ReforceTech AS, 3440 Røyken, Norway; len.miller@reforcetech.com; 6 Faculty of Science and Technology, The Arctic University of Norway, N-8505 Narvik, Norway; iveta.novakova@uit.no

**Keywords:** AR MiniBars, AR glass fiber, fly ash, geopolymer composites, mechanical properties

## Abstract

Geopolymer concrete reinforced with MiniBars™ could be an eco-friendly, innovative, durable, high-strength material substitute for common Portland cement in buildings. AR glass fiber MiniBars™ composites (AR MiniBars™) (ReforceTech AS, Royken, Norway) 60 mm in length were utilized to strengthen the geopolymer matrix for the fabrication of unidirectional geopolymer composites reinforced by AR MiniBars™ (AR MiniBars™ FRBCs). New AR MiniBars™ FRBCs were fabricated by adding different amounts of AR MiniBars™ (0, 12.5, 25, 50, 75 vol.%) into the fly ash geopolymer paste. Geopolymers were obtained by combining fly ash powder with Na_2_SiO_3_/NaOH in a ratio of 2.5:1, which served as an alkaline activator. AR MiniBars™ FRBCs were cured for 48 h at 70 °C and tested for different mechanical properties. Fly ash, AR MiniBars™, and AR MiniBars™ FRBC were evaluated by optical microscopy and SEM. The addition of AR MiniBars™ increased the mechanical properties of AR MiniBars™ FRBCs. The mechanical properties of AR MiniBars™ FRBCs were heightened compared to the geopolymer without AR MiniBars™; the flexural strength was 18.80–30.71 times greater, the flexural modulus 4.07–5.25 times greater, the tensile strength 3.49–8.27 times greater, the force load at upper yield tensile strength 3.6–7.72 times greater, and the compressive strength for cubic samples 2.75–3.61 times greater. The fractured surfaces and sections of AR MiniBars™ FRBCs were inspected by SEM and optical microscopy analyses, and even though there was no chemical adhesion, we achieved a good micromechanical adhesion of the geopolymer to AR MiniBars™. These results obtained encouraged us to propose AR MiniBars™ FRBCs for application in construction.

## 1. Introduction

Portland cement is one of the most utilized building materials, but its main problem is global CO_2_ emissions [1]. It contributes about 5% to global anthropogenic CO_2_ emissions [2]. Portland cement has certain disadvantages, like cracks, shrinkage, a slow setting time, limited resistance to chemical attacks, high energy use, and significant emission of greenhouse gas [3]. Replacing Portland cement with geopolymer concrete will improve the following parameters: energy use, costs, and negative environmental impact. The Portland cement production process consumes between 4000 and 4400 MJ of energy, 600 L of H_2_O, 800–900 kg of CO_2_, and 1 kg of SO_2_, and about 2 kg of NOx is produced [4]. On the other hand, geopolymer fabrication needs two times less energy, and around 4.5 times less CO_2_ is produced [4]. Geopolymers are inorganic polymers obtained from the dissolution of silicate and aluminate species with an alkaline solution (Na^+^, K^+^, Li^+^, Cs^+^, …) or acidic solution (phosphoric acid: H_3_PO_4_) and reorganization followed by the polycondensation reaction of aluminosilicates. The aluminosilicates used in a geopolymer could be kaolinite, feldspar, silica fume, red mud, calcined clays, zeolite, or industrial waste like fly ash, mining waste, waste glass, metallurgical slag, etc. [5].

Geopolymer concrete has presented comparable or even enhanced properties compared to common Portland concrete, including low energy use, a smaller carbon footprint, flame resistance, fire resistance, low permeability, good chemical resistance, good compressive strength, and good durability [6]. Despite poor mechanical properties, it could be a viable alternative to Portland cement, offering an environmentally friendly building material [7,8].

Geopolymers have shown depressed flexural and tensile strength because of their breakable ceramic texture [9], and this could limit their application. The addition of polypropylene fiber [10], glass fibers [11], basalt fibers [9,12], and steel fibers [13] to the geopolymers’ network could improve their physical-mechanical properties. Recently, our work showed that the addition of Basalt MiniBars™ to geopolymer composites increased the mechanical properties of the geopolymer: the flexural strength increased by 11.59–25.97 times, the flexural modulus by 3.33–5.92 times, the tensile strength by 3.50–8.03 times, and the force load at upper yield tensile strength by 4.18–7.27 times [9].

Generally, steel fibers are used in many building to reinforce concrete and provide improved mechanical properties, but, over time, this advantage could be lost, due to corrosion of the steel. The high alkalinity of Portland cement leads to corrosion of the glass-fiber-reinforced materials. The addition of a large amount of zirconia (15–20 wt.%) [14,15] to AR glass fibers provides a higher resistance to corrosion compared to other glass fibers [16]. Basalt fibers have a lower price than AR glass fibers, but the alkali-resistant character of the basalt fibers is less than that of AR glass fibers [17]. The polymer coating of the glass fiber surface showed substantially enhanced corrosion resistance to alkali degradation, tensile strength of alkali-resistant filament yarns, adhesion strength with cementitious matrices, and fracture energy of the composites [16]. MiniBars™ are impregnated with vinyl ester resin, offering them an advantage. Using chopped AR glass fibers in concrete could avoid drying shrinkage at early ages and increases the fracture toughness of the brittle matrix [18].

MiniBars™ are a high-performance alkali-resistant (AR) glass fiber or basalt fiber composite that provides advantages like improved post-cracking mechanical properties in hardened concrete, increased toughness, impact, and fatigue resistance in concrete, and lack of corrosion [19]. According to the ReforceTech AS Company, their product, MiniBars™, with AR glass fiber or basalt fibers, improves the performances of concrete by correcting the post-cracking mechanical properties of hardened concrete and improving the toughness, impact, and fatigue resistance of concrete, and through its lack of corrosion [19].

This study aimed to fabricate new unidirectional geopolymer composites reinforced with AR MiniBars™ (AR MiniBars™ FRBCs) with advanced mechanical properties and durability. AR MiniBars™ FRBCs were prepared by varying the volume of AR MiniBars™ between 0, 12.5, 25, 50, and 75 vol.% to reinforce the geopolymer paste. The filler used for developing geopolymers was fly ash powder from a coal power plant and was investigated by optical microscopy and scanning electron microscopy (SEM). AR MiniBars™ FRBCs were evaluated for mechanical behavior, including flexural strength, flexural modulus, tensile strength, tensile modulus, force load at upper yield tensile strength, and compressive strength. Also, the fractured and transversal section of AR MiniBars™ FRBCs specimens were analyzed by optical microscopy and scanning electron microscopy (SEM).

## 2. Materials and Methods

### 2.1. Materials

**Figure 1:**
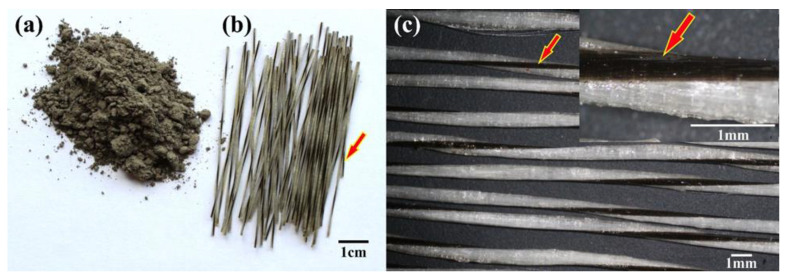
The images of (**a**) fly ash powder; (**b**,**c**) AR MiniBars™ (the red arrow shows the helical shape of AR MiniBars™).

Class F fly ash (Figure 1a) from a coal power plant (Mintia, Romania) was utilized to create a geopolymer matrix. Sodium hydroxide (NaOH) and sodium silicate (Na_2_SiO_3_) with modulus SiO_2_/NaOH = 2.5 were used as activators for fly ash powder to obtain geopolymers and were acquired from AGEXIM SRL, Romania. MiniBars™ (ReforceTech AS, Royken, Norway) (Figure 1b,c) are a chopped alkali-resistant (AR) glass-fiber-reinforced polymer (FRP) and are used for reinforcing the geopolymer matrix. AR MiniBars™ have a roving of 2400 AR fibers with an alkali-resistant (AR) glass fiber diameter of 19 µm, a 60 mm fiber length, and a 0.70 mm diameter of AR MiniBars™. AR fibers from MiniBars™ were impregnated with thermoset resin (vinyl ester resin) and had a helical shape and rough surface that offered micromechanical interlocking adhesion. According to the ReforceTech AS Company, the AR MiniBars™ had a total linear density of 888 tex (g/km) ± 5% and contained 68 wt.% AR glass fiber, 12 wt.% and 20 wt.% resin, and their density was around 2.1 g/cm^3^. The basalt helix gave the MiniBars™ their helical shape (Figure 1b,c, red arrow).

The composition of the fly ash was examined using a spectrofluorometer (JASCO FP-6500, Tokyo, Japan), which confirmed a 3.07 value for the SiO_2/_Al_2_O_3_ ratio in the fly ash, which, according to the classification, confirms the fly ash to be class F [19]. The size distribution of the fly ash was 0.103 μm at Dv50, the same as in previous work [19]. The degree of crystallinity of the fly ash and geopolymer was evaluated in previous work by X-ray diffraction [19]. The activator solution was a fresh, clear 14 M NaOH solution cooled to room temperature. Sodium silicate solution was combined with sodium hydroxide solution in a proportion of Na_3_SiO_3_/NaOH = 2.5:1.

### 2.2. Preparation of Unidirectional AR MiniBars™ FRBCs

The synthesis protocol for the preparation of geopolymer paste and AR MiniBars™ FRBCs has been reported in previous work [9]. The geopolymer paste was obtained by combining fly ash with the fluid Na_3_SiO_3_/NaOH at a weight ratio of 1.67. The homogeneous geopolymer paste was achieved by hand mixing for 10 min. AR MiniBars™ FRBC samples (n = 8) were prepared using a rectangular shape of Teflon (20 mm × 20 mm × 70 mm ± 0.1). The volume of a mold filled lengthwise with AR MiniBars™ had weight 22.66 g. Based on these volumes vs. weight, we obtained AR MiniBars™ FRBCs with different volumes of AR MiniBars™ (Table 1). Samples were prepared in the mold with the geopolymer paste by adding the AR MiniBars™ into the geopolymer paste. AR MiniBars™ were pressed into the geopolymer paste and moved down, up, and sideways with a stick in the paste so that they were wetted with the geopolymer paste. To eliminate the air bubbles from the samples, a vibrating table was used for 5 min, and the mold was pressed with a plastic film and glass plate and the sample was cured at 70 °C for 48 h. The samples were detached and checked and those with voids were excluded from the study. The cubic samples (20 mm × 20 mm × 20 mm) (n = 8) for the compressive strength test were prepared from a transversal section of the rectangular samples (20 mm × 20 mm × 70 mm ± 0.1) prepared above.

### 2.3. Optical Microscopy and Scanning Electron Microscopy (SEM)

The fly ash, the AR glass fiber, and the surfaces of the AR MiniBars™ FRBC samples after the FS test were also investigated using a stereomicroscope (Stemi 2000-C, Carl Zeiss, AG, Oberkochen, Germany). The sample morphology of the fly ash, AR MiniBars™, and AR MiniBars™ FRBCs and the structure of the fractured surfaces of the AR MiniBars™ FRBC samples were analyzed using a stereomicroscope (Stemi 2000-C, Carl Zeiss AG, Germany) as well as SEM (SEM Inspect S, FEI, The Netherlands).

### 2.4. Mechanical Characterization of AR MiniBars™ FRBCs

The mechanical properties (flexural strength, flexural modulus, tensile strength, tensile modulus, and force load at upper yield tensile strength) of AR MiniBars™ FRBCs were evaluated using a universal testing machine (LR5K Plus, Lloyd instruments. Ltd., Bognor Regis, UK) at the speed of 1 mm/min.

The flexural strength (FS) was determined from Equation (1).

FS = 3F_max_l/2bh^2^ (MPa) (1)

where F_max_ is the applied force (N), l is the distance between the supporting anvil (50 mm), b is the width (20 mm), and h is the thickness (20 mm). The flexural modulus (MPa) was calculated from the slope in the elastic portion of a stress–strain curve obtained by the flexural test.

The tensile strength (TS) was determined from Equation (2).

TS = F/(A) (MPa) (2)

where F is the applied force (N) on the cross-section of the sample at tension. A is the cross-sectional area of the sample (mm^2^).

The tensile modulus (MPa) was calculated from the slope in the elastic portion of a stress–strain curve obtained by tensile test. The force load at upper yield tensile strength (kN) was registered as the maximum force in the elastic area during the tensile test for the AR MiniBars™ FRBCs.

The compressive strength (CS) values for cubic samples (20 mm × 20 mm × 20 mm) were determined using Equation (3). For this test, we used a universal testing machine (LBG srl, Azzano San Paolo (BG), Italy) at the speed of 10 mm/min. The force applied to the samples was parallel with the AR MiniBars™ alignment in the samples.

CS = F/A (MPa) (3)

where F is the applied load (N), A is the cross-sectional area of the cubic sample (mm^2^).

## 3. Results

**Figure 2:**
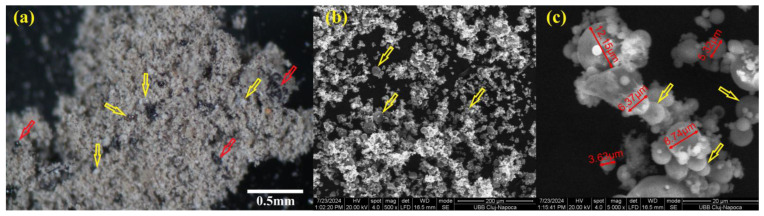
(**a**) Optical microscopy of fly ash powder; (**b**,**c**) SEM micrographs of fly ash (red arrows indicate black and non-spherical particles, yellow arrows indicate spherical particles).

According to our previous work [9,20], the white spherically shaped particles observed by optical microscopy were Mulite [21] (Figure 2a, yellow arrow), and the black spherical/non-spherical particles were Hematite–Fe_2_O_3_ (Figure 2a, red arrow). Spherical particles can also be seen in SEM images (Figure 2b,c, yellow arrow) and were also specified in other studies [9,20,21,22,23]. The XRD analysis of fly ash from previous work [20,22] showed Quartz–SiO_2_ (PDF#461045), Mullite–3Al_2_O_3_·SiO_2_ (PDF#150776), and Hematite–Fe_2_O_3_ (PDF#330664) in a small quantity. The fly ash showed that the spherical/non-spherical vitreous phase was together with the crystalline one [20]. The alkali hydroxide solution NaOH/Na_2_SiO_3_ activator was used to mix with the fly ash powder to dissolve the surface of the aluminosilicate network structure from fly ash and to release active ions, such as SiO_4_^4−^, AlO_4_^5−^, Mg^2+^, and Ca^2+^, etc., used for the polycondensation process.

**Figure 3:**
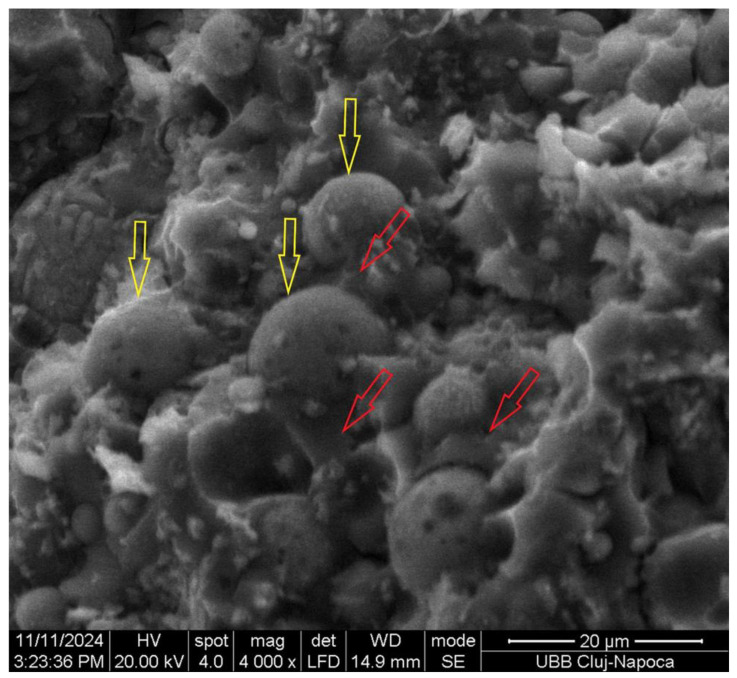
SEM micrographs of the surface of fractured geopolymer (yellow is a spherical particle, the red arrow is geopolymer).

All these cations will be able to reorganize, followed by a polycondensation reaction of aluminosilicates, and to form new bonds and sialate (Si-O-Al-O) chained structures. This geopolymer matrix covers spherical/non-spherical particles (Figure 3, yellow arrow) of fly ash and contributes to the strengthening of the material, which agreed with other publications [20,22,23,24].

**Figure 4:**
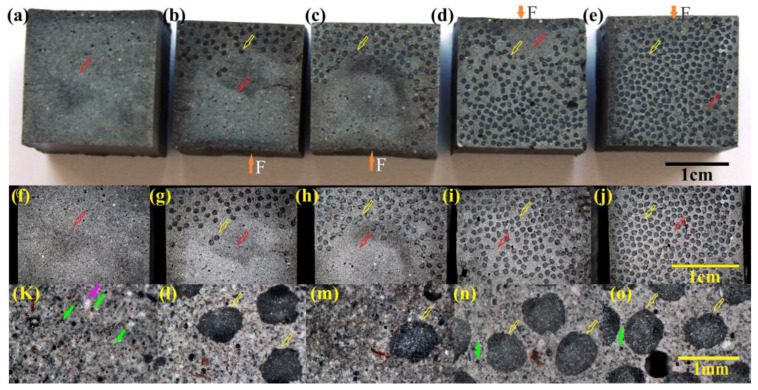
The images of the sections of AR MiniBars™ FRBCs: (**a**) Fly 100; (**b**) AR MiniBars12.5; (**c**) AR MiniBars25; (**d**) AR MiniBars50; and (**e**) AR MiniBars75. Optical images of the sections of AR MiniBars™ FRBCs: (**f**,**k**) Fly 100; (**g**,**l**) AR MiniBars12.5; (**h**,**m**) AR MiniBars25; (**i**,**n**) AR MiniBars50; and (**j**,**o**) AR MiniBars75 (orange arrow shows force applied; yellow arrow indicates AR MiniBars™; red arrow indicates geopolymer; green arrow indicates black non-spherical particles; pink arrow indicates a spherical particle).

In Figure 4, the geopolymer section and AR MiniBars™ distribution on a transversal section of an AR MiniBars™ FRBC rectangular sample can be seen. The number of black dots (yellow arrow) increases when the AR MiniBars™ quantity increases from 12.5 to 75 vol. %, as shown in Figure 4b–e,g–j. In samples with 12.5 and 50 vol. %, as shown in Figure 4b,c,g,h, the fiber distribution exceeds in one part of the sample. This can be explained by the pressing and vibration mode of the sample during preparation. Around the fibers, the geopolymer distribution (gray color, red arrow) can be seen. The optical images in Figure 4l–o show more details about the shape and distribution of the AR MiniBars™. The image from Figure 4k shows also black and white spherical particles, which can also be observed in Figure 2. The flexural strength test was performed with contact that involved tipping (force) up the samples removed from the mold, as shown in Figure 4b–e by the orange arrow. This means the fiber density was an opposite force for AR MiniBars25, AR MiniBars50, and AR MiniBars75, with the exception of AR MiniBars75 when the fiber distribution was homogeneous. This difference in the fiber distribution was because we used the hand pressure of AR MiniBars during preparation and also the vibration table was used during the preparation of samples.

**Figure 5:**
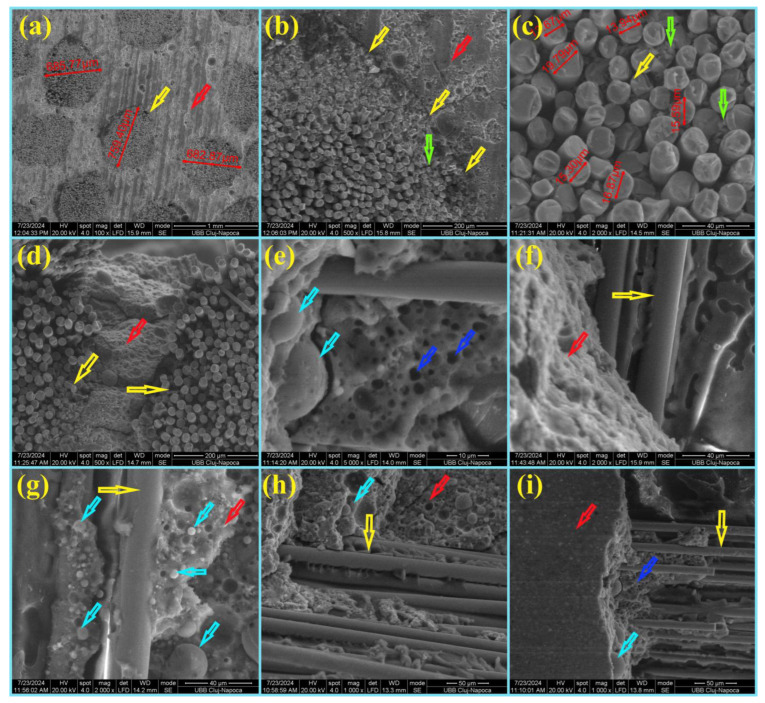
SEM images of AR MiniBars™ FRBCs: (**a**–**c**) transverse section of AR MiniBars75 after flexural test; (**d**–**i**) fracture of AR MiniBars75 after flexural test (yellow arrow indicates AR MiniBars™; red arrow indicates geopolymer; green arrow indicates black thermoset resin; turquoise arrow indicates a spherical particle; blue arrow indicates air bubbles).

Figure 5a shows SEM images of the transversal section of AR MiniBars™ (yellow arrow) and the geopolymer around them. AR MiniBars™ do not have a regular shape, being surrounded by the geopolymer, which adheres properly on them. Figure 5b shows in more detail the interface between the AR MiniBars™ (yellow arrow) and the geopolymer (red arrow). Also, the diameter of the AR glass fiber (Figure 5b,c) from the roving and some thermoset resin (green arrow) coating the AR glass fiber and entering between the fibers is shown. Figure 5d–i detail the fracture of AR MiniBars75 after the flexural test, and the AR glass fibers from AR MiniBars™ (yellow arrow) at the interface with the geopolymer (red arrow) can be seen. In Figure 5e,g,i, the turquoise arrow points to the geopolymer around spherical particles from the fly ash, which can also be observed in Figure 2. During the preparation of samples, vibration was used to remove the air from the geopolymer paste. Unfortunately, some air remained in some micropores, as shown in Figure 5e,i (blue arrow).

**Figure 6:**
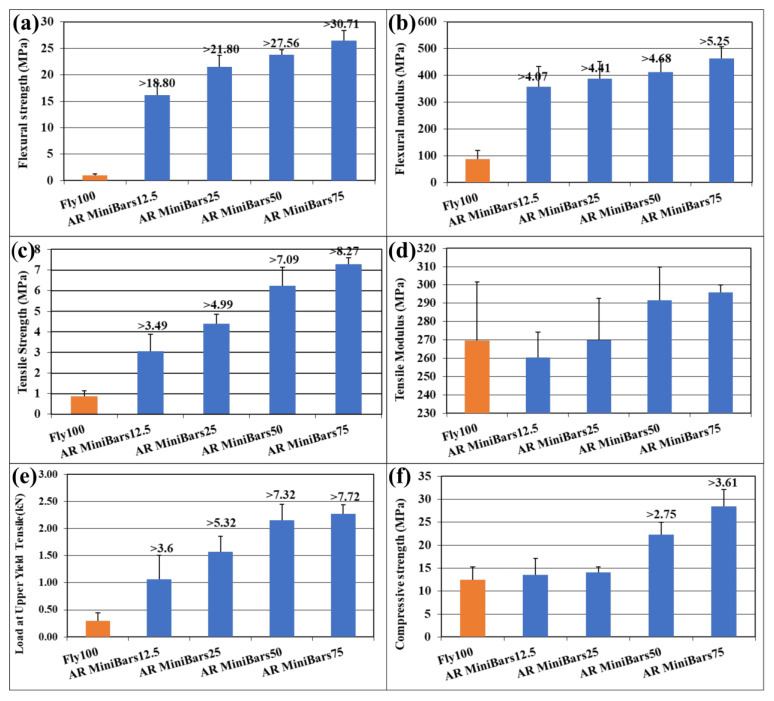
Mechanical properties of AR MiniBars™ FRBCs: (**a**) flexural strength; (**b**) flexural modulus; (**c**) tensile strength; (**d**) tensile modulus, (**e**) force load at upper yield tensile strength; (**f**) compressive strength.

Mechanical testing values of AR MiniBars™ FRBCs (Figure 6) generally showed higher values than samples of geopolymer without AR MiniBars™ fibers. This supported increasing the reinforcing amount of AR MiniBars™. The samples of AR MiniBars75 showed the highest flexural strength, flexural modulus, tensile strength, tensile modulus, and force load at upper yield tensile strength. The flexural strength values of AR MiniBars™ FRBC (Figure 6a) were between 16.20 and 26.47 MPa, which means they were more than 18.80–30.71 times that of Fly100. The flexural strength results of AR MiniBars™ FRBCs were well correlated with the quantity of AR MiniBars™ (y = 3.3005x + 10.442, R^2^ = 0.958) in the composition. The same behavior was observed for the flexural modulus of AR MiniBars™ FRBC (Figure 6b), with values between 358.38 and 462.61 MPa, which very well correlated with the increasing quantity of AR MiniBars™ (y = 33.625x + 287.57, R^2^ = 0.9715). The highest increase for the flexural strength and the flexural modulus, 18.80 times and 4.07 times greater than those of the geopolymer, was for AR MiniBars12.5. After the addition of AR MiniBars™, there were not such large differences between samples. 

**Figure 7:**
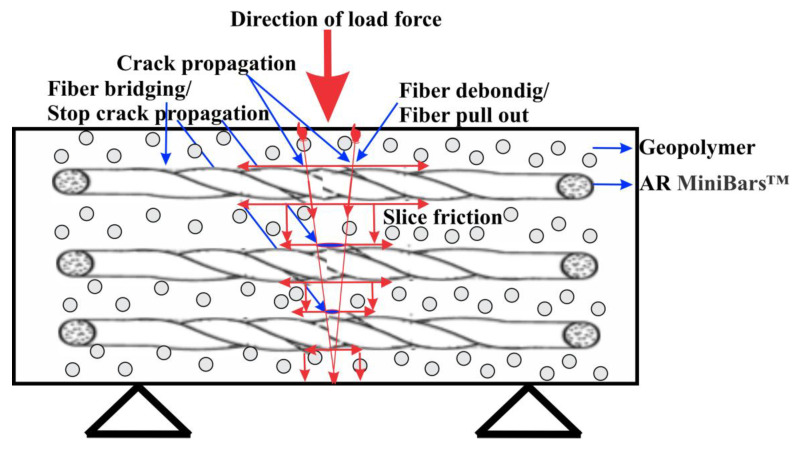
Schematic image of the crack propagation on a sample in the flexural strength test.

The continuous unidirectional AR MiniBars™ had an orientation parallel with the length of the mold (Figure 7). When a force is applied on AR MiniBars™ FRBCs, perpendicular to the fiber orientation, the AR MiniBars™ at the first level in the geopolymer will break and the other AR MiniBars™ will act as crack stoppers. If the force increases, the AR MiniBars™ will try to resist this force, but, at the same time, some events will appear, such as fiber debonding, slice compression, and fiber pulling out, and the last fiber will break when the crack reaches the next level of the AR MiniBars™. When the indenter presses the sample in the flexural test, the geopolymer and AR MiniBars™ will deform, by sliding the region between the fibers and increasing the pressure from slice to slice (Figure 7). In the structure of the geopolymer (Figure 7), we could see many spherical particles fixed in the 3D matrix of the geopolymer binder. The surface of AR MiniBars™ was created for this type of micromechanical adhesion and is similar to the surface of a deformed steel bar used in construction. Unfortunately, at the surface of AR MiniBars™, a chemical bond, it is not possible, but the helix surface of AR MiniBars™ will be an advantage for micromechanical adhesion. There are two internal changes inside the sample when the load is applied. First, an elastic deformation occurs, which is a reversible deformation, and, at the end, a plastic deformation occurs, which is irreversible and will generate cracks and the failure of the geopolymer and AR MiniBars™ or the pull-out of the AR MiniBars™ from the geopolymer. In the flexural test, the crack propagation in the samples appears in the direction of the applied force.

An anisotropic mechanical property was observed when the direction of the force applied was the same as the orientation of the fibers, and when the highest stiffness and strength were registered [25]. The orientation of the fibers in the composite will provide different mechanical properties. According to Krenchel’s reinforcing factor [26], the chopped fibers give the 3D randomly oriented short fibers in composites a strengthening factor of 0.2, whereas 2D-oriented fibers (woven) give 0.375 and unidirectional fibers give a factor of 1.

The tensile strength of AR MiniBars™ FRBCs (Figure 6c) showed values between 3.07 and 7.28 MPa, more than 3.49–8.27 times those of Fly100 (y = 1.4495x + 0.1715, R^2^ = 0.9895), and the tensile modulus (Figure 6d) was in the range of 260.51–295.82 MPa (y = 12.749x + 234.81, R^2^ = 0.9346). For this test, the helix shape of AR MiniBars™ is an advantage if the chemical bond is missing. In our opinion, the geopolymer broke before the AR MiniBars™ during this test. Inside the sample, a break of the geopolymer between AR MiniBars™, a break of the AR MiniBars™, or the pull-out of AR MiniBars™ could occur. The tensile strength test was also influenced by the compression of the geopolymer in the pneumatic sample clamping system and could decide the maximum tensile strength. With the limit of our test, we considered the geopolymer around AR MiniBars™ to be broken before the AR MiniBars™. In our opinion, the tensile test registered the failure of the geopolymer around the fiber in the middle of the sample and not the AR MiniBars™. More investigation with microCT or other non-destructive investigation of the inside of the sample is required. The force load at upper yield tensile strength of the AR MiniBars™ FRBCs (Figure 4e) was between 1.06 and 2.27 kN, more than 3.6–7.72 times that of Fly100 (y = 0.4967x, R^2^ = 0.9938). 

If we compare all these results with our previous results when we used basalt MiniBars™ [9], we can observe that the results for the mechanical properties (Table 2) were very close to those of this study. The flexural strength of AR MiniBars™ showed the greatest difference from basalt MiniBars™. Based on these results, from our point of view, AR MiniBars™ could be more advantageous, because they have ZrO_2_ content that offers them a higher resistance to corrosion than that of basalt MiniBars™.

The compressive strength for cubic samples (Figure 6f) showed values between 13.51 and 28.45 MPa, with a difference 2.75–3.61 times higher for AR MiniBars50 and AR MiniBars75, respectively. This test is very important in practice when the force is applied in the length of the support element for different applications in construction. AR MiniBars50 and AR MiniBars75 showed the positive effect of the AR MiniBars™ reinforcement. 

AR MiniBars™ replace in deformed steel bars many applications. This is due to the non-corroding nature and cost effectiveness of the MiniBars-reinforced concrete. MiniBars™ FRBCs could be a very promising sustainable building material and a real alternative to Portland cement. MiniBars permit the structural engineer to use thinner concrete (or FRBC) due to the elimination of the typical cover layer needed to protect the steel and allow a sacrificial layer for steel fibers. A typical steel rebar solution with 100 kg/m^3^ can be solved with 10 kg/m^3^ of MiniBars. The MiniBars with a density that is a quarter of that of steel lead to a very high fiber count, and also the 3D uniform dispersion of the MiniBars, including at the surface, thus ensures that the entire cross-section is acting to strengthen the concrete or FRBC. The documented CO_2_ savings of MiniBar-reinforced concrete result in 20 to 50% reductions through the use of basalt or AR MiniBars, reduction/elimination of the steel used and less concrete. The same savings would be found when using a more sustainable FRBC. The savings are documented in hundreds of business cases for specific projects over the last decade, as demonstrated by the customers using MiniBars [27].

## 4. Conclusions

The mechanical properties of AR MiniBars™ FRBCs increased with an increased number of AR MiniBars™ with the following parameters: the flexural strength increased by 18.80–30.71 times, the flexural modulus by 4.07–5.25 times, the tensile strength by 3.49–8.27 times, the force load at upper yield tensile strength by 3.6–7.72 times, and the compressive strength for cubic samples by >2.75–3.61 times. The test showed a lesser increase in tensile modulus with the addition of AR MiniBars™. Optical images and SEM images showed a good adhesion of the geopolymer to the AR MiniBars™. AR MiniBars™ replace some applications for deformed steel bars, which are corrosive and more expensive. AR MiniBars™ FRBCs could be a very promising sustainable building material and a real alternative to Portland cement.

## Figures and Tables

**Table 1:** Composition of the AR MiniBars™ FRBCs.

Nr.	Code	vol. % AR MiniBars™	wt. % AR MiniBars™
1	Fly100	0	0
2	AR MiniBars12.5	12.5	2.83
3	AR MiniBars25	25	5.66
4	AR MiniBars50	50	11.33
5	AR MiniBars75	75	16.99

**Table 2:** Range of mechanical properties of 12.5, 25, 50, 75 vol.% basalts vs. AR MiniBars™ FRBCs.

Nr.	Mechanical Properties	Basalt MiniBars™	AR MiniBars™
1.	The flexural strength (MPa)	9.99–22.39	16.20–26.47
2.	The flexural modulus (MPa)	267.74–475.63	358.38–462.61
3.	Tensile strength (MPa)	3.08–7.06	3.07–7.28
4.	Tensile modulus (MPa)	292.58–341.68	260.51–295.82
5.	Force load at upper yield tensile strength (kN)	1.23–2.14	1.06–2.27

## Data Availability

The data that support the findings of this study are contained within the article.
